# Tolerance of High-Dose Oral Amiodarone for Cardioversion of Atrial Flutter

**DOI:** 10.1016/j.cjco.2022.04.006

**Published:** 2022-04-28

**Authors:** Vincent K. Le, Katherine M. Kavanagh, Satish R. Raj, P. Timothy Pollak

**Affiliations:** a Department of Cardiac Sciences, Cumming School of Medicine, University of Calgary, Calgary, Alberta, Canada; b Libin Cardiovascular Institute, Cumming School of Medicine, University of Calgary, Calgary, Alberta, Canada; c Department of Medicine, Cumming School of Medicine, University of Calgary, Calgary, Alberta, Canada

## Abstract

In atrial arrhythmias, amiodarone is usually given either intravenously for acute management, requiring in-hospital monitoring, or orally for chronic control, as doses given 60 times per half-life, requiring weeks to reach full effect. A high-risk, 245-kg male with heart failure exacerbated by atrial flutter was successfully cardioverted using an atypically large, 8000-mg oral amiodarone dose. The only adverse effect was transient sinus arrest, which did not require intervention, only 24 hours of inpatient monitoring. Amiodarone’s unique pharmacokinetics, including its long elimination half-life and its extensive distribution into a large volume of adipose tissue, make high-dose oral amiodarone boluses a reasonable strategy for cardioversion of atrial arrhythmias.

## Résumé

En présence d’arythmie auriculaire, l’amiodarone est généralement administrée par voie intraveineuse dans la phase aiguë de la prise en charge, ce qui nécessite une surveillance du patient en milieu hospitalier, ou encore par voie orale dans le cadre d’un traitement au long cours à des doses représentant 60 fois la demi-vie, le plein effet du médicament n’étant obtenu qu’au bout de plusieurs semaines. Un homme de 245 kg à haut risque souffrant d’insuffisance cardiaque aggravée par un flutter auriculaire a subi avec succès une cardioversion médicamenteuse faisant appel à une dose exceptionnellement élevée d’amiodarone – 8000 mg – administrée par voie orale. Le seul effet indésirable a été une pause sinusale n'ayant pas nécessité d'intervention, seulement 24 heures de surveillance en milieu hospitalier. Vu la pharmacocinétique particulière de l’amiodarone, notamment sa longue demi-vie d’élimination et sa distribution étendue dans un grand volume de tissu adipeux, l’administration perorale de ce médicament en dose de charge constitue une stratégie raisonnable de cardioversion en cas d’arythmie auriculaire.

Intravenous amiodarone, but not oral amiodarone, is commonly used for cardioversion of atrial fibrillation. This is likely because a single 200-mg oral tablet is such a small fraction of the total dose usually administered during the drug’s ∼60-day half-life and because slow absorption over 12 hours following ingestion blunts and delays peak concentration compared to intravenous administration. Nonetheless, single oral bolus doses of amiodarone at 30 mg/kg have been shown to be safe in cardioverting atrial arrhythmias.^1^^,^^2^

Pharmacologic principles suggest that each person has a threshold concentration, above which effective cardioversion becomes a high probability. Given sufficient time for absorption, a sufficiently large oral dose should produce a concentration above that threshold. A systematic review of several small studies of high-dose oral amiodarone for cardioversion suggests that it is effective.^3^

**Figure 1:**
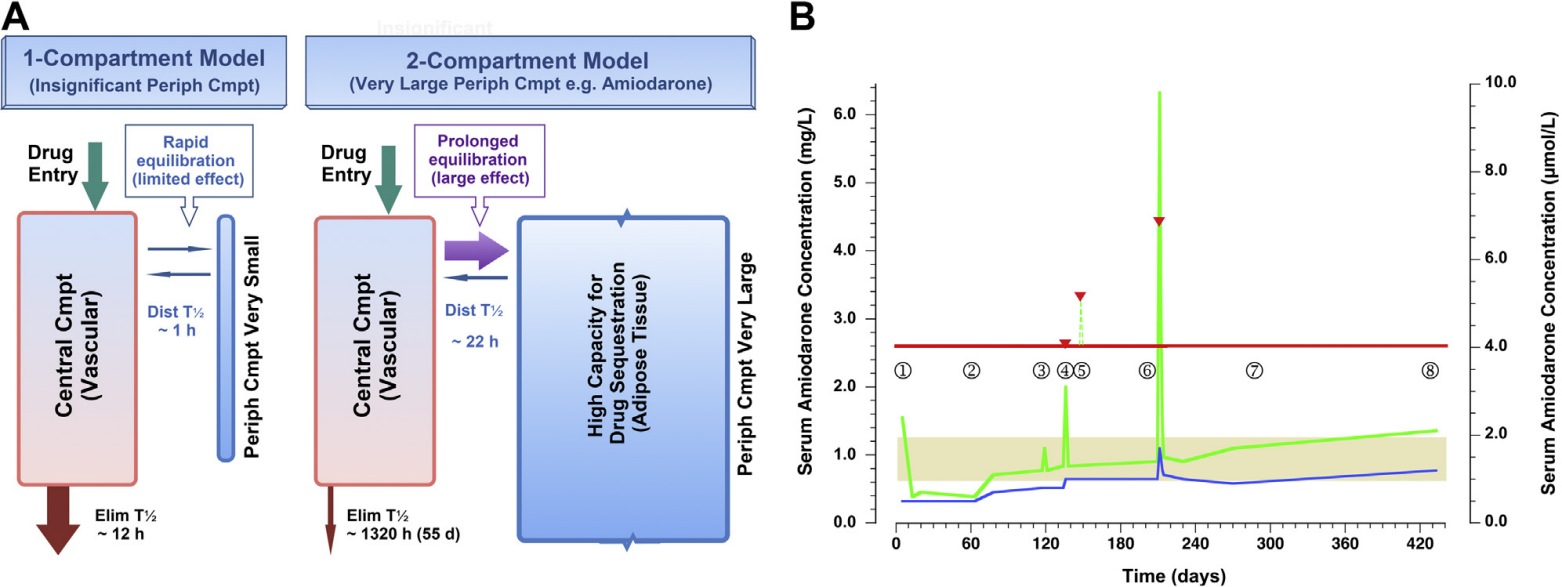
(**A**) Two-compartment (cmpt) model of amiodarone pharmacokinetics. Most medications conforming to a 2-cmpt model have the following: a small peripheral (Periph) cmpt (considered 1-cmpt if very small); an elimination half-life (Elim T½) measured in hours; a steady state reached within days; and vascular concentrations that are highly sensitive to multiples of a normal daily dose. Amiodarone instead has the following: a very large periph cmpt; a T½ of distribution (Dist) rapid enough to promote net movement of drug out of the vascular cmpt until concentrations in periph cmpt slowly equilibrate to steady state (**purple arrow**); and vascular concentrations that, following any rapid change, return to near pre-bolus values by distributing drug to periph cmpts. (**B**) Serum concentrations of amiodarone (AMIO) during 14 months of therapy. The **green line** indicates AMIO; the **blue line** indicates desethylamiodarone (DEA) metabolite; the **yellow bar** indicates desirable amiodarone range for long-term maintenance; the **red line** indicates target threshold for local transient AMIO concentration to effect rhythm conversion; the **red triangles** indicate predicted 12-hour AMIO concentration post 4800-, 6000-, and 8000-mg oral boluses, based on first 2000-mg reading. **①** Day 5—transient AMIO elevation from loading doses falls rapidly with distribution out of serum, once slower maintenance dosing is started. **②** Day 63—AMIO and DEA slowly increase during ∼ 430 mg/d dosing rate. **③** Day 118—oral bolus of 2000 mg gives only a transient AMIO bump and returns to baseline with distribution. **④** Day 135—oral bolus of 4800 mg (20 mg/kg) gives lower than predicted AMIO spike. **⑤** Day 149—oral bolus of 6000 mg unsuccessful; AMIO sample missed by patient; maintenance dose increased to 460 mg/d. **⑥** Day 210—oral bolus of 8000 mg (30 mg/kg) successful cardioversion, but AMIO concentration 30% higher than predicted; transient nodal blockade resolves rapidly as AMIO falls with redistribution; start maintenance dosing of 400 mg/d. **⑦** Day 270—periph cmpt saturating on maintenance dosing of 400 mg/d; AMIO and DEA concentrations rising. **⑧** Day 433—sinus rhythm still preserved; AMIO at top end of desirable range; maintenance dose lowered to 300 mg/d to avoid further accumulation.

We used this information, guided by measurement of 12-hour post-dose amiodarone concentrations, to help address concerns encountered in a patient with atypically high body mass index (BMI), for whom anaesthetic risks excluded electrical cardioversion. Instead, we sought cardioversion using an oral medication that did not require in-hospital monitoring. Given his BMI, published weight-based dosing seemed excessive, but initial attempts with smaller doses failed. Only higher weight-based dosing produced cardioversion, and the patient tolerated high single-dose amiodarone exposure, as predicted by the drug’s relatively rapid distribution out of blood (Fig. 1A and B).

## Case

**Figure 2:**
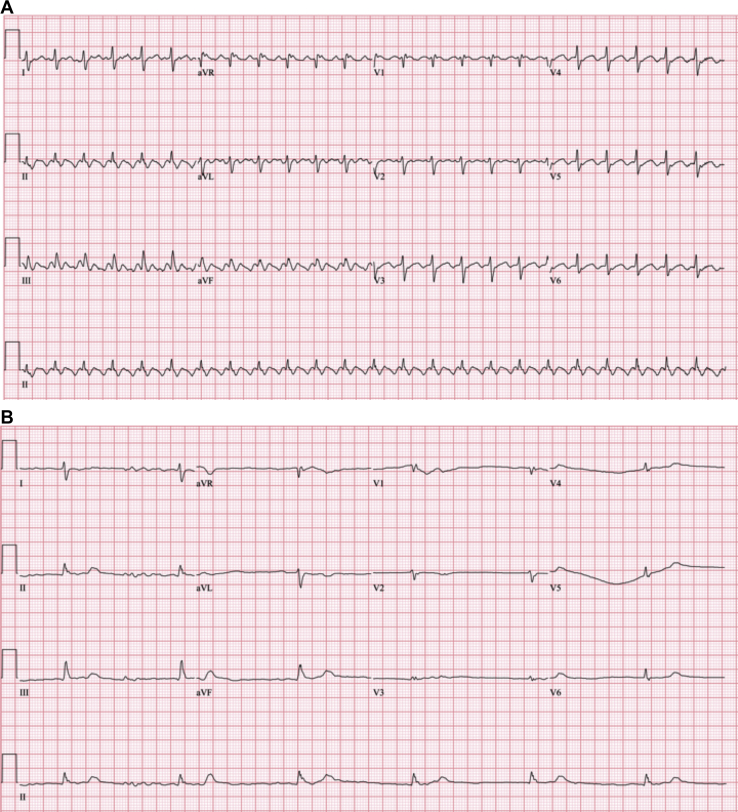
Electrocardiograms (ECGs) before and after pharmacologic cardioversion. (**A**) Initial ECG prior to cardioversion showing typical anticlockwise atrial flutter with 2:1 conduction, heart rate of 145 beats per minute, and rightward axis. (**B**) ECG post-cardioversion at presentation to the hospital showing sinus arrest with a junctional escape rhythm, heart rate of 36 beats per minute, and rightward axis.

A 67-year-old man presented with worsening dyspnea on exertion, and electrocardiogram revealed new atrial flutter at a heart rate (HR) of 145 beats per minute (bpm; Fig. 2A). At 245 kg (540 lb), his BMI was 66 kg/m^2^, resulting in hypoxia from obesity-hypoventilation syndrome and obstructive sleep apnea requiring home bilevel positive airway pressure (BiPAP) treatment. His medications included candesartan 8 mg/d, rosuvastatin 20 mg/d, insulin glargine 48 units twice daily, and furosemide 40 mg as needed. An echocardiogram showed severe right-ventricular dysfunction, normal left-ventricular systolic function, and no significant valvular disease. Apixaban 5 mg twice daily was initiated, in addition to metoprolol uptitrated to 100 mg twice daily. The dosage was then lowered due to presyncope and hypotension. A trial of digoxin was discontinued for inefficacy for a heart rate of 120 bpm. While continuing metoprolol titrated from 75 mg down to 25 mg twice daily, oral amiodarone was initiated at 400 mg twice daily for 10 days (Fig. 1B, day 0), followed by 400 mg/d. Two months later, he remained dyspneic with atrial flutter at 120 bpm, which made pursuing cardioversion highly desirable. However, the safety of electrical cardioversion was limited by serious anaesthetic risks and bariatric equipment limitations. Outpatient high-dose oral amiodarone, shown to be safe for converting atrial arrhythmias,^4^ was chosen to avoid the need for hospital admission associated with standard electrical or intravenous pharmacologic cardioversion.

Based on previous clinical experience with oral amiodarone cardioversion, an initial 2000-mg oral dose was administered (Fig. 1B, day 118). A 12-hour post-administration serum amiodarone concentration was only 1.6 μmol/L (1.0 mg/L), and Holter monitoring confirmed uninterrupted atrial flutter at 120 bpm. Based on a threshold of ∼4.0 μmol/L (2.5 mg/L), below which toxicity is less likely during chronic dosing.^5^ Our goal was to attain this value. The next evening, a repeat 2000-mg dose was also unsuccessful, and amiodarone concentration was not collected. Following a 16-day interval (day 134), another oral bolus was attempted at 20 mg/kg (4800 mg), and 12-hour post-dose serum amiodarone concentration reached only 3.0 μmol/L (1.9 mg/L), with no resolution of atrial flutter (day 135). Two weeks later, a dose of 6000 mg produced no change in rhythm. The patient failed to present for serum amiodarone concentration assessment.

Aggressive maintenance dosing was continued for another 2 months to improve saturation of the peripheral compartment. On day 209, the published 30 mg/kg (8000 mg) dose ^2^ was taken in the evening, and the patient awoke feeling well, with a regular heart rate of 60 bpm on a Holter monitor. However, when he presented to the phlebotomy lab to provide a sample for amiodarone concentration measurement, he felt light-headed, and his heart rate was 32 bpm (day 210). Serum amiodarone concentration was found to be 9.8 μmol/L (6.3 mg/L). The Holter monitor confirmed a gradual slowing of his atrial flutter, evolving into ventricular pauses reaching 4.1 seconds (Fig. 2B) followed by sinus arrest with a junctional escape rhythm at 37 bpm. This prompted admission for monitoring.

Over the next several hours, sinus rhythm returned at 50 bpm, and he was discharged after 24 hours of cardiac monitoring. At 21 hours and 54 hours after initial sampling, respectively, his serum amiodarone concentration was found to be 4.9 μmol/L (3.2 mg/L) and 2.2 μmol/L (1.4 mg/L), confirming that the half-life of distribution between the vascular and peripheral compartments was 21 hours. An oral maintenance dose of amiodarone 200 mg twice daily was prescribed, and he remained in sinus rhythm at each follow up. An electrocardiogram at 10 months post discharge confirmed sinus bradycardia at 46 bpm, similar to his rate prior to atrial flutter, and his serum amiodarone concentration was 2.1 μmol/L (1.4 mg/L). Repeat echocardiogram showed only mild right-ventricular dysfunction.

## Discussion

Amiodarone’s superior efficacy reflects its broad range of antiarrhythmic actions across all 4 Vaughan Williams Classifications,^6^ its consistent presence in cardiac tissue, and the narrow diurnal variation in serum drug concentration that result from its extremely long elimination half-life.^7^

### Pharmacokinetics of oral amiodarone

The pharmacokinetics of amiodarone are best represented by a 2-compartment model— central/vascular and peripheral/adipose (Fig. 1A)—with rapid inter-compartmental distribution.^7^ These characteristics explain the success of using a single high-dose amiodarone bolus to produce transient elevations in concentration sufficient for cardioversion, while maintaining safety, even at a single oral dose of 30 mg/kg. This success is due to its half-life of distribution between the 2 compartments (15-20 hours) being ∼80 times shorter than its elimination half-life, causing it to be quickly distributed away from the vascular compartment. As a result, bolus amiodarone entering the central (vascular) compartment, from an intravenous or gastrointestinal source, only transiently elevates the serum concentration. Without further doses, the vascular compartment quickly equilibrates with the peripheral/adipose compartment, returning the vascular compartment to near pre-dose serum concentrations and avoiding prolonged high-concentration exposure sufficient to cause toxicity.

Dosing amiodarone daily is a pattern quite different than that used for other drugs, as revealed by the typical dose per half-life (TDPH) metric. Whereas for many commonly used drugs, such as metoprolol, the administered dose (25 mg) given once per half-life is also the TDPH, daily dosing of amiodarone at 200 mg, is actually “micro-dosing” at 1/60th of its 12,000-mg TDPH, being administered every 1/60th of its ~60-day half-life. Despite the safety and efficacy of giving a full TDPH once every half-life for shorter half-life drugs, doing so with amiodarone would not be practical, pleasant, or clinically acceptable. However, given that the toxicity of amiodarone occurs with high concentration exposure over time, rather than with a single, rapid exposure, a single TDPH would not be fatal if spaced one half-life from another similarly large dose. Given amiodarone’s pharmacokinetics, smaller fractions of TDPH administered as a single dose should be commensurately safer, as seen by the lack of any life-threatening effects or cardiac arrhythmias in patients who have attempted self-harm in taking up to 8000 mg of amiodarone.^8^

### Managing oral amiodarone in this case

Measuring serum amiodarone concentration following the 6000-mg oral bolus (Fig. 1B, day 149) would have permitted a better estimate of the suitability of the subsequent 8000-mg dose. In Figure 1B, the measured 12-hour post-dose concentrations did not follow a linearly proportional pattern with increases in dosing, most likely reflecting saturation of inter-compartmental distribution at high doses, and increased tissue saturation. Thus, although the post-8000-mg concentration was effective, it produced unexpectedly high concentrations. This was associated with transient sinus arrest and junctional escape rhythm, before it rapidly dissipated by half over 21 hours. Management of the patient entailed only observation, did not require temporary pacing, or other interventions, and he had no evidence of organ toxicities. The benefits to the patient were improvement of heart failure after achieving sinus rhythm and avoidance of the anaesthetic risk of electrical cardioversion. After many months, the patient remains stable in sinus rhythm on an oral amiodarone maintenance dose, without recurrence of his atrial flutter and with his dose eventually reduced to 200 mg/d based on slowly rising serum amiodarone concentrations.

The advantages of oral amiodarone over in-hospital cardioversion are similar to those of other out-of-hospital strategies^4^ including the oral drug being much less expensive, its adverse-effect profile being more favourable compared to intravenous administration, and the fact that it does not require the expense of hospital admission. Most patients (with a more typical BMI) would not present the same difficulty in estimating an optimal oral converting dose.

## Conclusion

The unique pharmacokinetics of amiodarone, including its relatively short distribution time, compared to its long elimination half-life, predict that administration of a single large oral dose will be a safe and effective method for pharmacologic cardioversion of atrial arrhythmias. Measurements of serum amiodarone concentration can be a useful adjunct to guide the dosing of oral amiodarone in this setting.

**Novel Teaching Points**

• A single large oral dose of amiodarone can produce a transient elevation in amiodarone concentration that is safe, efficacious, and cost-effective in cardioverting atrial arrhythmias.
• Pharmacologic cardioversion of atrial arrhythmias with oral amiodarone is a suitable option for outpatient therapy.
• Measuring serum amiodarone concentration may help guide safe and efficacious dosing of amiodarone.
